# Prevalence and risk factors of potentially inappropriate medications at discharge in older adults with heart failure: A retrospective observational pharmacovigilance study

**DOI:** 10.1097/MD.0000000000049387

**Published:** 2026-06-19

**Authors:** Junfen Xu, Liqin Zhang, Hailiang Ma, Yuanben Lu, Zhenhua Jiang

**Affiliations:** a Department of Cardiology, Shaoxing Central Hospital, Shaoxing, Zhejiang, China.

**Keywords:** Beers Criteria, discharge transition, heart failure, potentially inappropriate medications

## Abstract

Older adults with heart failure (HF) are at high risk of potentially inappropriate medications (PIMs) during hospital discharge, yet real-world data from Asian populations remain limited. This study aimed to investigate the prevalence, spectrum and predictors of PIMs at discharge among older patients with HF in Eastern China. This retrospective cross-sectional study enrolled 468 patients aged ≥ 65 years with chronic HF hospitalized between 2022 and 2024. PIMs were identified according to 2023 American Geriatrics Society Beers Criteria. The primary outcome was PIM prevalence, and predictors were analyzed using multivariable logistic regression. PIMs are highly prevalent in older patients with HF at discharge. Polypharmacy, atrial fibrillation (AF) and renal impairment are the main risk factors. Targeted medication reconciliation and electronic medical record-based alerts are needed to reduce preventable drug-related harm. The overall prevalence of PIMs at discharge was 87.4% (409/468), with a mean of 2.4 ± 1.8 PIM instances per patient. Three independent predictors were identified: discharge medication count ≥ 7 (aOR = 3.45, 95% confidence interval (CI): 2.10–5.66), AF (adjusted odds ratio [aOR] = 2.98, 95% CI: 1.89–4.68), and eGFR < 45 mL/min/1.73m^2^ (aOR = 1.74, 95% CI : 1.05–2.88). A synergistic interaction between AF and polypharmacy further elevated PIM risk. Common PIMs included furosemide, rabeprazole and rivaroxaban.

## 1. Introduction

Heart failure affects > 10% of individuals aged ≥ 70 years globally.^[1]^ In China, the 2023 Cardiovascular Health Report documented 8.9 million heart failure (HF) patients, 62% aged ≥ 65 years – a 40% increase over the past decade.^[2]^ Eastern China exemplifies this shift, with the elderly population exceeding 20% and HF hospitalizations rising 7.3% annually.^[3]^ The discharge transition emerges as a high-risk period characterized by medication intensification and fragmented monitoring.

Potentially inappropriate medications (PIMs), defined by the 2023 American Geriatrics Society (AGS) Beers Criteria, represent drug–patient dyads where harm exceeds benefit. PIM exposure correlates with 30% excess readmission rates and 1.8-fold increased mortality risk within 6 months post-discharge. These risks are amplified in older adults by age-related pharmacokinetic alterations and pharmacodynamic hypersensitivity.^[4–6]^

HF patients exhibit unique PIM susceptibility: multimorbidity is ubiquitous (75% with CCI ≥ 3), mandating polypharmacy (median 8–12 discharge medications) that escalates drug–drug interaction (DDI) risk exponentially. Renal dysfunction (eGFR < 60 mL/min/1.73m^2^) affects 45% of this cohort, yet renally cleared medications are frequently under-dosed. Atrial fibrillation (AF), present in 35% to 50%, obligates anticoagulation and antiarrhythmic use, yet Beers Criteria flag amiodarone and non-dose-adjusted rivaroxaban as high-risk.^[7,8]^

While PIM prevalence among general older outpatients is well-characterized (25–70% in North America and Europe), data specific to the HF discharge transition are conspicuously absent. Existing U.S. studies report 53% PIMs in ambulatory HF clinics, while Chinese investigations are confined to outpatient cohorts, yielding rates of 62% to 70% but overlooking the high-risk discharge window. This gap is critical: medication regimens intensify by an average of 2.3 drugs during hospitalization, yet discharge reconciliation remains suboptimal.^[7,9]^

We conducted this study to quantify PIM prevalence at discharge among older patients with HF in Eastern China. We hypothesized PIM prevalence would exceed 80%, reflecting polypharmacy and disease complexity. Secondary aims were to identify independent predictors, with a priori hypotheses that AF and discharge medication burden ≥ 9 drugs would emerge as synergistic risk factors, and that renal impairment (eGFR < 45 mL/min/1.73m^2^) would independently amplify PIM risk.

## 2. Methods

### 2.1. Study design and setting

We conducted a retrospective pharmacovigilance study nested within the Cardiology Department of Shaoxing Central Hospital, a 1600-bed tertiary academic center designated as a National Heart Failure Center by the National Health Commission of China. This center’s integrated EMR and pharmacy system with automated WHO ATC coding represents a best-case scenario that may not reflect resource-limited settings. Consecutive admissions between January 1, 2022, and December 31, 2024, were screened.

#### 2.1.1. Ethics statement

This study was approved by the Ethics Committee of Shaoxing Central Hospital (approval number: 2025-069). The study was conducted in accordance with the Declaration of Helsinki. Informed consent was waived due to the retrospective design and use of anonymized patient data.

### 2.2. Participants

Inclusion criteria: age ≥ 65 years; primary discharge diagnosis of chronic HF documented by International Classification of Diseases, 10th revision codes I50.0–I50.9 (validated by 2 cardiologists); hospitalization duration ≥ 48 hours; and survival to discharge with complete pharmaceutical records.

Exclusion criteria: in-hospital death, transfer, active malignancy, solid organ transplantation, or missing data.

Sample size calculation: Based on preliminary audit data (n = 50) indicating 85% PIM prevalence, PASS 2021 estimated that 400 patients would provide 95% power to detect an adjusted OR of 2.0 at α = 0.05. However, pilot samples often overestimate true prevalence. To ensure robustness, we recalculated using a conservative 70% prevalence estimate (based on published Chinese outpatient data, which required n = 420 to maintain 95% power.^[10,11]^

### 2.3. Data collection

Baseline variables were extracted by research assistants (inter-rater agreement κ = 0.95): demographics, cardiac function (New York Heart Association [NYHA] class, left ventricular ejection fraction, N-terminal pro-B-type natriuretic peptide), comorbidities (CCI),^[12]^ laboratory parameters (eGFR, electrolytes, hemoglobin), and hospitalization metrics (length of stay, cardiac care unit (CCU) admission, 30-day readmission history).

Medication data were extracted from discharge orders and processed through a custom R script to map generic names to WHO ATC codes and calculate total discharge medication count.^[13]^ Discharge medications were stratified into tertiles (1–4, 5–8, ≥9 drugs) based on clinical relevance and sample size distribution. Guideline-directed HF therapies (angiotensin receptor-neprilysin inhibitor, ACE inhibitors/ angiotensin receptor blockers, β-blockers, mineralocorticoid receptor antagonists (MRAs), SGLT2 inhibitors, ivabradine) were identified.^[14]^

The standard CCI’s mandatory HF weight (+3) induces multicollinearity (VIF = 2.8). We therefore pre-specified a modified CCI (excluding HF weight) for sensitivity analysis.^[15]^

### 2.4. Outcome ascertainment

The primary outcome – presence of ≥1 PIM – was adjudicated through a rigorous two-step clinical validation process to avoid overestimation and ensure contextual appropriateness.

Step 1: An R script automatically screened discharge medications against the 2023 AGS Beers Criteria to generate an initial list of potential PIMs, including drugs in the “Avoid,” “Use with Caution,” renal dose adjustment, DDI, and HF-specific contraindication domains.

Step 2: Two senior clinical pharmacists independently reviewed all flagged cases (not just a sample) by comprehensively evaluating clinical context, including documented indications, dosage, treatment duration, renal function, age, comorbidities, and therapeutic necessity. Drugs were only classified as PIMs if the potential harm exceeded benefit in the specific clinical context of each patient. Inter-rater reliability was high (κ = 0.86, n = 200 randomly selected cases). Any disagreements were resolved by a senior geriatrician specializing in geriatric pharmacotherapy. This manual clinical review ensured that automated screening results were refined by real-world clinical judgment, minimizing inappropriate overclassification of necessary medications as PIMs.^[16]^

### 2.5. Statistical analysis

Descriptive statistics were reported as mean ± standard deviation, median (interquartile range [IQR]), or frequencies (%). Univariate screening (*P* < .10) identified variables for multivariable logistic regression examining PIM exposure (age, sex, AF, eGFR < 45 mL/min/1.73m^2^, NYHA ≥ III, Charlson ≥ 3, medication count ≥ 7, CCU stay). Model robustness was assessed via variance inflation factor (VIF) and Hosmer–Lemeshow test; discrimination by area under the curve. Linear trends across medication quartiles used Cochran–Armitage test. Pre-specified sensitivity analyses excluded: CCU admissions > 48 hours; eGFR < 30 mL/min/1.73m^2^; and prior 30-day readmissions. Stratified analyses by age and AF tested interactions. Significance set at *P* < .05 (two-sided) using SPSS 29.0.

## 3. Results

### 3.1. Patient flow and baseline characteristics

**Figure 1.**
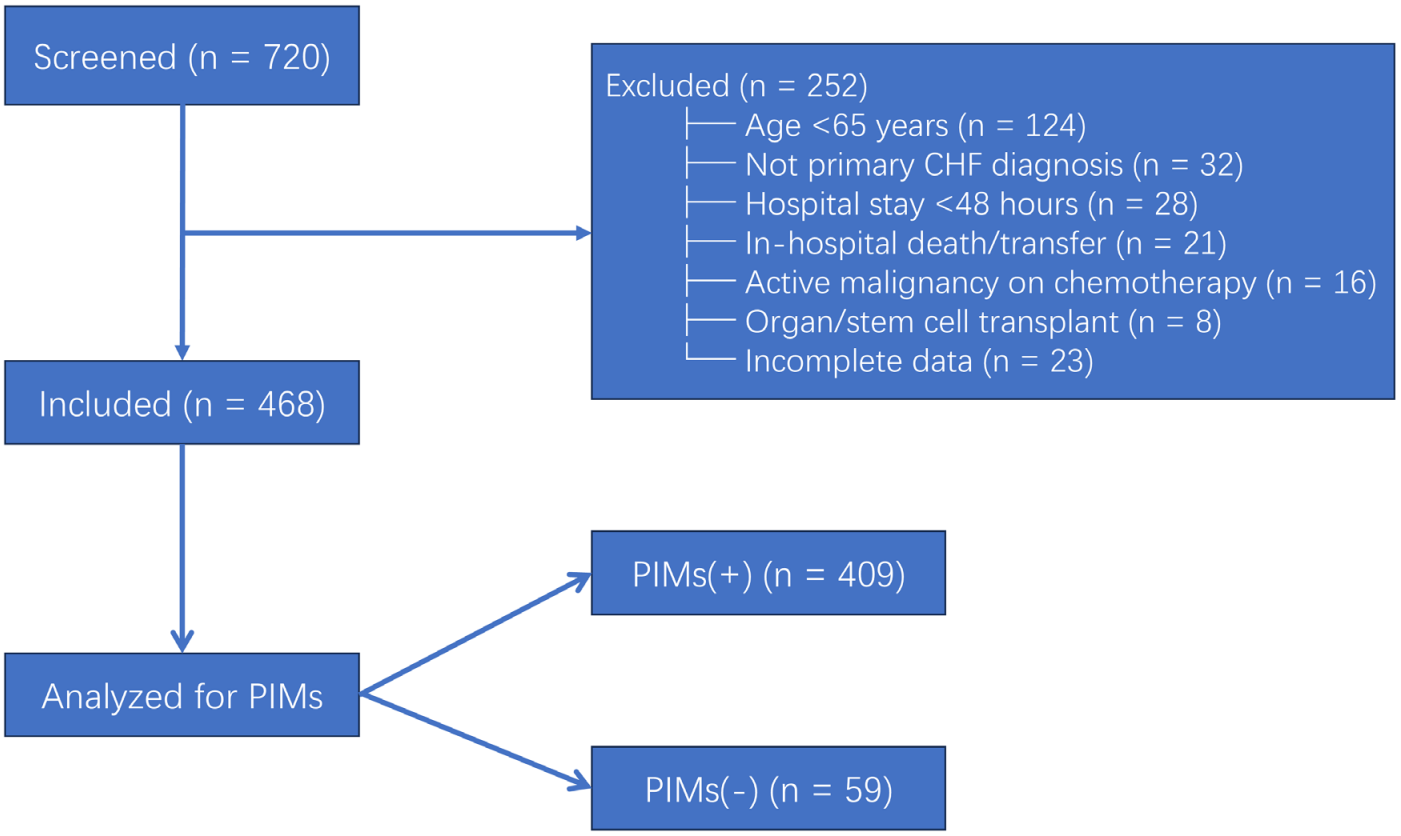
CONSORT flow diagram of study participants.

During the three-year study period (January 1, 2022, to December 31, 2024), we screened 720 consecutive admissions with a primary diagnosis of HF at Shaoxing Central Hospital. After excluding 252 individuals who met predefined criteria – most commonly age < 65 years (n = 124) and non-CHF primary diagnosis upon clinical adjudication (n = 32) – 468 older adults with confirmed chronic HF were included in the final analytic cohort, representing a 65.0% inclusion rate (Fig. 1).

Comparative analysis identified significant baseline disparities between PIM-positive and PIM-negative cohorts (Table 1). Patients with PIMs (n = 409, 87.4%) were significantly older (*P* < .001) and demonstrated a higher burden of AF (46.2% vs 22.0%, *P* < .001). While sex distribution and body mass index were comparable, the PIM-positive group exhibited more advanced cardiac dysfunction, evidenced by lower left ventricular ejection fraction (*P* = .015) and elevated N-terminal pro-B-type natriuretic peptide levels (*P* = .006). Comorbidity burden was also greater in the PIM-positive group, with a median CCI of 4 (IQR 3–5) versus 3 (IQR 2–4) (*P* = .008). Notably, rates of guideline-directed medical therapy were equivalent between groups (renin-angiotensin system inhibitors 62.6% vs 64.4%, *P* = .454; β-blockers 76.3% vs 74.6%, *P* = .442), while 30-day readmission history showed a marginal difference (21.3% vs 11.9%, *P* = .060). Laboratory parameters, including renal function, were broadly similar across cohorts.

**Table 1:** Baseline characteristics of older adults with chronic heart failure by PIMs status.

Characteristic	PIMs(+) (n = 409)	PIMs(−) (n = 59)	*P*-value
Demographics			
Age, yr	78.1 ± 6.1	74.0 ± 5.3	<.001
Male sex, n (%)	238 (58.2)	38 (64.4)	.223
BMI, kg/m^2^	24.3 ± 3.9	24.8 ± 4.3	.313
Employee medical insurance, n (%)	271 (66.3)	41 (69.5)	.369
Cardiac function			
NYHA class, n (%)			
I	22 (5.4)	3 (5.1)	.349
II	128 (31.3)	25 (42.4)	
III	195 (47.7)	25 (42.4)	
IV	64 (15.6)	6 (10.6)	
LVEF, %	40.6 ± 10.9	44.2 ± 9.5	0.015
NT-proBNP, pg/mL	4548 ± 2883	3487 ± 1925	0.006
Comorbidity burden			
Charlson Comorbidity Index, median	4 (3, 5)	3 (2, 4)	.008
Atrial fibrillation, n (%)	189 (46.2)	13 (22.0)	<.001
Diabetes mellitus, n (%)	81 (19.8)	8 (13.6)	.168
Coronary artery disease, n (%)	235 (57.5)	32 (54.2)	.370
Cardiomyopathy n (%)	73 (17.8)	9 (15.3)	.390
Valvular heart disease n (%)	32 (7.8%)	3 (5.1%)	.331
Hypertension, n (%)	286 (69.9)	38 (64.4)	.237
COPD, n (%)	68 (16.6)	8 (13.6)	.352
Peripheral vascular disease, n (%)	37 (9.0)	5 (8.5)	.560
Cancer, n (%)	11 (2.7)	3 (5.1)	.254
Laboratory parameters			
eGFR, mL/min/1.73 m^2^	66.9 ± 32.4	69.1 ± 30.1	.624
Serum sodium, mmol/L	138.2 ± 4.1	138.8 ± 3.3	.240
Serum potassium, mmol/L	4.2 ± 0.5	4.1 ± 0.4	.097
Hemoglobin, g/L	124.7 ± 18.0	128.1 ± 21.2	.190
Hospitalization metrics			
Length of stay, d	9 (7, 13)	8 (6, 11)	.125
CCU admission > 48 h, n (%)	48 (11.7)	4 (6.8)	.183
30-d readmission history, n (%)	87 (21.3)	7 (11.9)	.060

BMI = body mass index, CCU = cardiac care unit, e-GFR = estimated glomerular filtration rate, LVEF = left ventricular ejection fraction, NT-proBNP = N-terminal pro-B-type natriuretic peptide, NYHA = New York Heart Association, PIM = potentially inappropriate medication.

### 3.2. Dose-dependent relationship between polypharmacy and PIM burden

**Figure 2.**
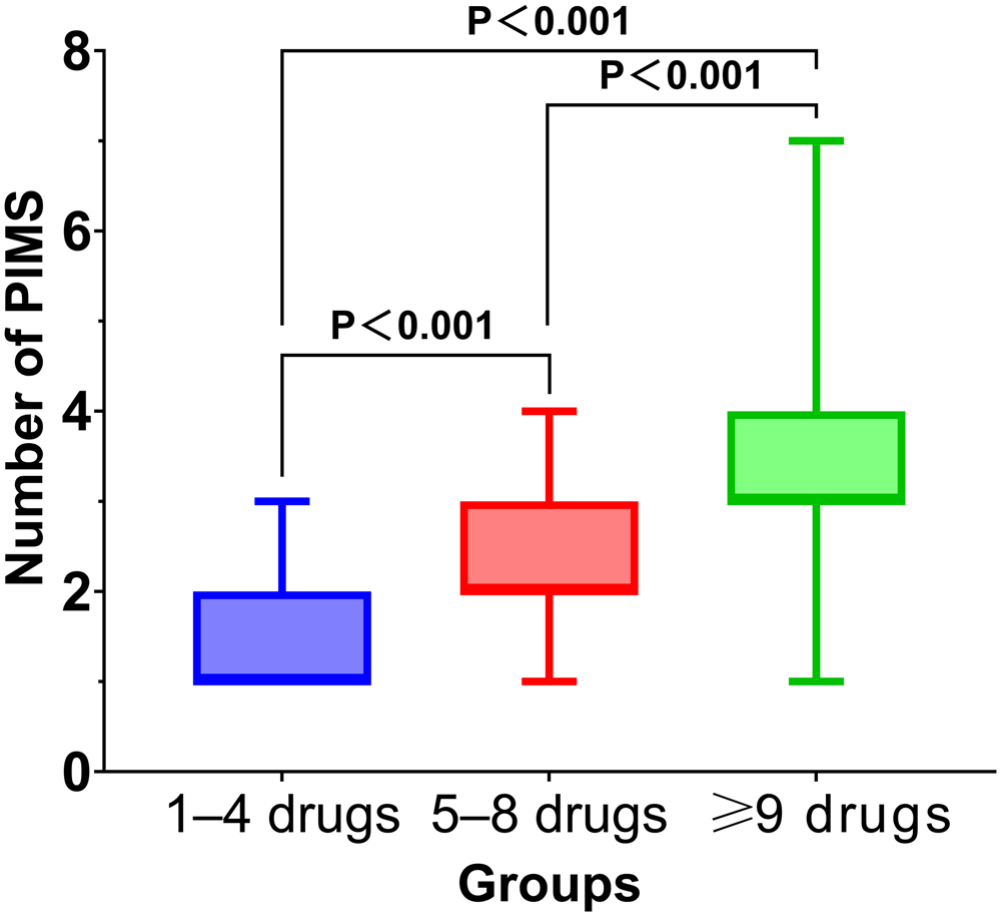
Dose-dependent relationship between discharge medication count and PIM burden. PIM = potentially inappropriate medication.

A significant dose-dependent relationship existed between discharge medication count and PIM burden (Table 2 and Fig. 2). The PIM-positive group had a markedly higher mean medication count than the PIM-negative group (8.6 ± 5.6 vs 5.5 ± 1.6, *P* < .001). Distribution analysis revealed a strong gradient: among patients prescribed 1 to 4 drugs, only 8.1% of the PIM cohort fell in this range versus 32.2% of the PIM-negative group. Conversely, nearly half of PIM-positive patients (49.4%) received ≥ 9 medications compared with just 6.8% of those without PIMs (*P* < .001). Notably, PIM prevalence reached 91.9% in the high-burden (≥9 drugs) stratum, establishing polypharmacy as the principal driver of inappropriate prescribing. Importantly, usage rates of guideline-directed medical therapies (renin-angiotensin system inhibitors, β-blockers, MRAs, and SGLT2 inhibitors) were comparable between groups (all *P* > .05).

**Table 2:** Comparison of discharge medication patterns between groups.

Variable	PIMs(+) (n = 409)	PIMs(−) (n = 59)	*P*-value
Discharge medication count	8.6 ± 5.6	5.5 ± 1.6	<.001
Discharge medication distribution, n (%)			
1–4 drugs	33 (8.1)	19 (32.2)	<.001
5–8 drugs	174 (42.5)	36 (61.0)	
≥9 drugs	202 (49.4)	4 (6.8)	
GDMT usage, n (%)			
RAAS-i	256 (62.6)	38 (64.4)	.454
β-blocker	312 (76.3)	44 (74.6)	.442
MRA	184 (45.0)	27 (45.8)	.510
SGLT2-i	104 (25.4)	15 (25.4)	.571

GDMT = guideline-directed medical therapy, MRA = mineralocorticoid receptor antagonist, PIM = potentially inappropriate medication, RAAS = renin-angiotensin system, SGLT2i = sodium-glucose cotransporter 2 inhibitor.

**Figure 3.**
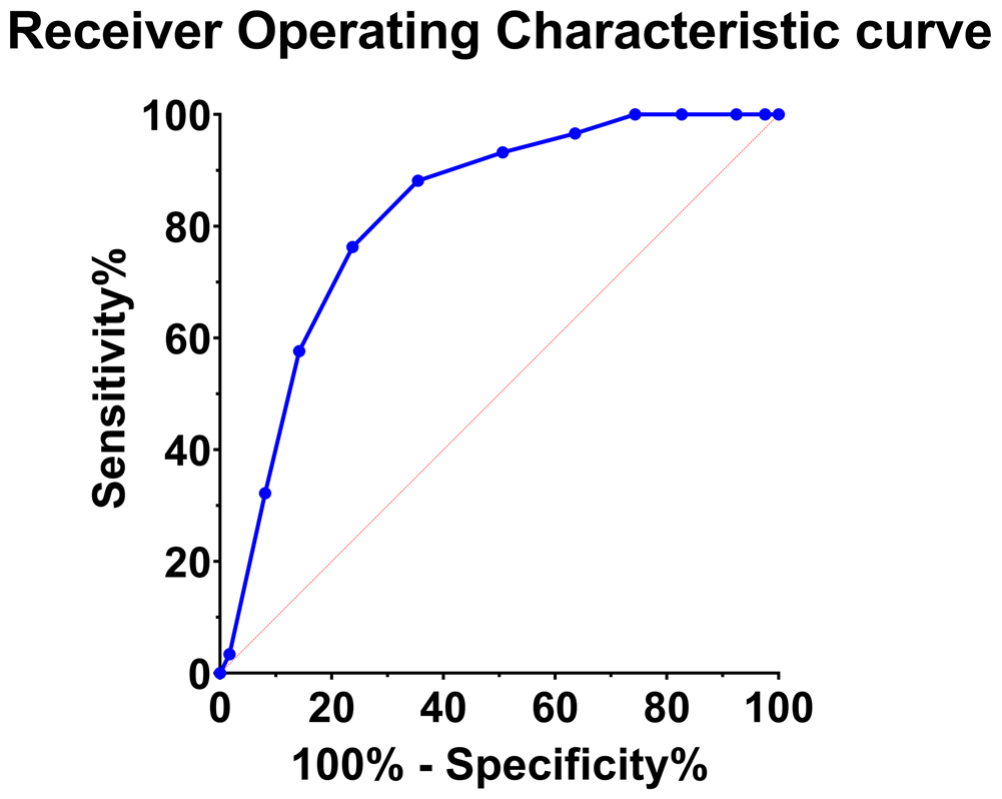
ROC curve for optimal polypharmacy threshold identification. ROC = receiver operating characteristic.

To determine the optimal polypharmacy threshold for predicting PIM risk, receiver operating characteristic curve analysis evaluated cutoffs from 1 to 13 medications. The optimal cutoff was identified at 7 medications (area under the curve = 0.82, 95% confidence interval (CI): 0.77–0.87, *P* < .001), maximizing Youden index (0.53) with sensitivity 88.1% and specificity 64.5% (Fig. 3). This statistically-derived threshold aligns with the observed risk inflection where PIM probability exceeds 85% (Fig. 3).

### 3.3. Spectrum and classification of PIMs

Among the 468 eligible patients, 409 (87.4%) had at least one PIM at discharge, with 1008 total instances documented (mean 2.4 ± 1.8 per patient). Table 3 enumerates the 15 most frequent individual PIMs, accounting for 72.3% of all instances. Loop diuretics predominated in the Use with Caution category, with furosemide prescribed in 213 patients (21.2%). Proton pump inhibitors represented the leading Avoid class,^[17]^ with rabeprazole (n = 89, 8.8%) and esomeprazole (n = 43, 4.3%) frequently lacking documented indication. Among anticoagulants, rivaroxaban (n = 52, 5.2%) was flagged for both renal dose omission and DDIs, while spironolactone (n = 47, 4.7%) appeared in the Renal Dose Adjustment domain, with 68.1% failing dose reduction despite eGFR < 45 mL/min.^[18]^ Notable Avoid medications included amiodarone (n = 42, 4.2%) and lorazepam^[19]^ (n = 31, 3.1%).

**Table 3:** Top 15 individual potentially inappropriate medications identified at discharge.

Rank	Drug (ATC-5)	Beers domain	Numbers	Primary rationale (2023 AGS Beers)
1	Furosemide	Caution	213	Hyponatremia/dehydration risk in older adults
2	Rabeprazole	Avoid	89	PPI > 8 wk without indication; C. difficile risk
3	Empagliflozin	Caution	52	Genital infection & volume depletion
4	Rivaroxaban	Avoid + DDI	52	eGFR 30–50 mL/min dose omission; bleeding with amiodarone
5	Spironolactone	Renal	47	eGFR < 45 mL/min hyperkalemia risk
6	Esomeprazole	Avoid	43	As above (PPI)
7	Amiodarone	Avoid	42	Long-term toxicity > thyroid/pulmonary/falls
8	Lorazepam	Avoid	31	Falls, delirium, cognitive impairment
9	Diltiazem	HF-specific	28	Negative inotropy in HFrEF
10	Glibenclamide	Avoid	25	Severe prolonged hypoglycemia
11	Cilostazol	HF-specific	24	Increased mortality in HF
12	Edoxaban	Renal	23	60 mg vs 30 mg in eGFR 15–29
13	Dapagliflozin	Caution	22	As empagliflozin
14	Digoxin	Caution	20	Serum level > 1.2 ng/mL or eGFR < 50
15	Insulin (basal)	Caution	18	Sliding scale without basal optimization

AGS = American Geriatrics Society, DDI = drug–drug interaction, e-GFR = estimated glomerular filtration rate, HF = heart failure, HFrEF = heart failure with reduced ejection fraction.

**Figure 4.**
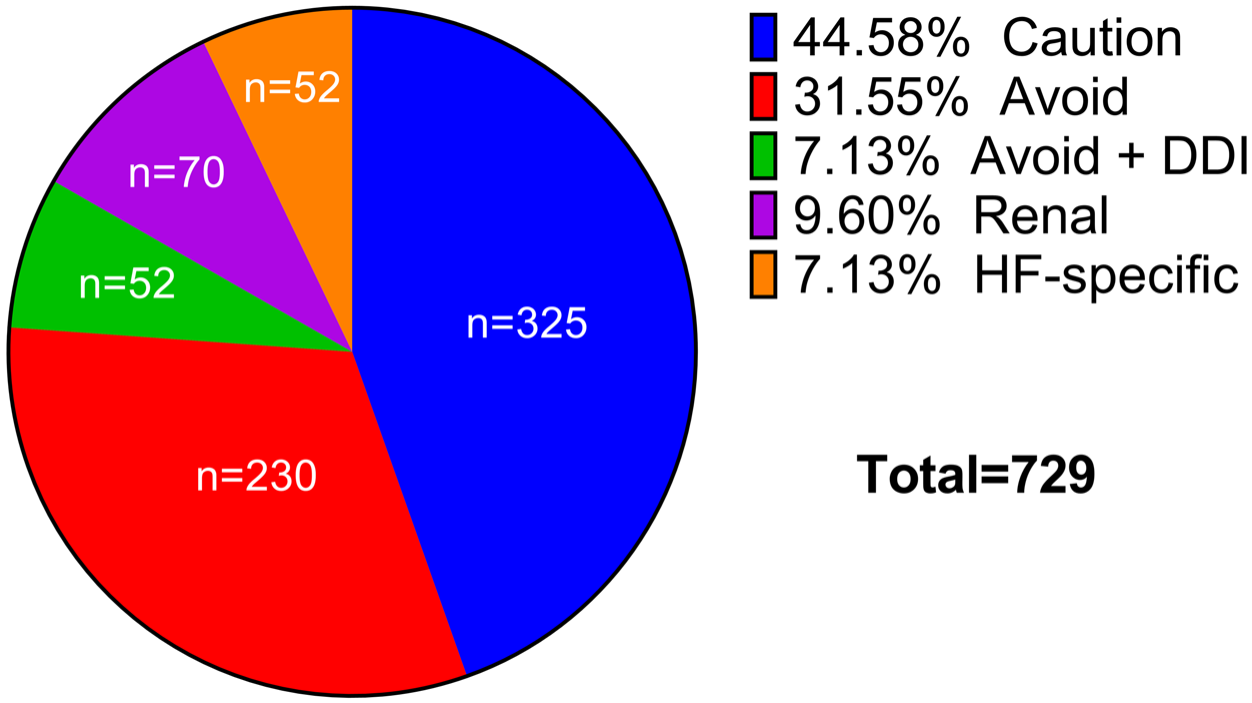
Distribution of Beers domain in top 15 individual potentially inappropriate medications.

Figure 4 depicts the Beers domain distribution within these top 15 drugs. Across the full cohort, 325 instances were classified as Avoid, 230 as Use with Caution, 52 as drug–drug Interactions, 70 as Renal Dose Adjustment, and 52 as HF–specific contraindications.

### 3.4. Independent predictors of PIMs

Multivariable logistic regression identified 3 independent predictors of PIM exposure after adjustment for twelve clinically relevant covariates (Table 4). The final model demonstrated excellent fit and absence of multicollinearity (VIF range 1.2–2.1). AF emerged as the strong clinical predictor (adjusted OR: 2.98, 95% CI: 1.89–4.68, *P* < .001), followed by discharge medication burden ≥ 7 drugs (adjusted OR: 3.45, 95% CI: 2.10–5.66, *P* < .001). Renal impairment (eGFR < 45 mL/min/1.73 m^2^) independently increased PIM risk by 74% (adjusted OR: 1.74, 95% CI: 1.05–2.88, *P* = .031). Notably, age, sex, NYHA class, and modified Charlson index ≥ 3 were not independently associated with PIMs in the multivariable context.

**Table 4:** Multivariable logistic regression analysis of PIMs risk factors.

Variables	β	SE	*P*-value	aOR	95% CI	VIF
Age	0.086	0.093	.356	1.09	0.91–1.31	2.1
Male sex	−0.236	0.297	.424	0.79	0.44–1.41	1.3
NYHA class III–IV	0.285	0.256	.264	1.33	0.81–2.20	2.4
Atrial fibrillation	1.092	0.231	<.001	2.98	1.89–4.68	1.8
Modified CCI ≥ 3	0.512	0.201	.011	1.67	1.12–2.48	1.4
Discharge medications ≥ 7	1.238	0.253	<.001	3.45	2.10–5.66	1.6
CCU stay > 48 h	0.166	0.329	.620	1.18	0.62–2.24	1.2
RAAS-i	−0.139	0.257	.592	0.87	0.53–1.44	1.9
β-blocker	−0.051	0.260	.838	0.95	0.57–1.58	2.3
SGLT2-i	0.199	0.242	.408	1.22	0.76–1.96	1.4
Renal impairment	0.554	0.256	.031	1.74	1.05–2.88	1.8

aOR = adjusted odds ratio, CCI = Charlson Comorbidity Index, CCU = cardiac care unit, CI = confidence interval, NYHA = New York Heart Association, PIM = potentially inappropriate medication, RAAS = renin-angiotensin system, SGLT2i = sodium-glucose cotransporter 2 inhibitor, VIF = variance inflation factor.

Table 5 demonstrates a significant interaction (*P*-interaction = .031) between AF and polypharmacy. In patients without AF, those prescribed ≥ 7 drugs had an absolute PIM risk of 65.2% (vs 31.5% with <7 drugs), yielding a 2.10-fold adjusted odds ratio. In contrast, AF patients with >8 drugs faced a 94.2% absolute risk (vs 39.5% with <7 drugs), resulting in a substantially higher adjusted OR of 5.12. The risk difference was +33.7% in non-AF patients versus +54.7% in AF patients.

**Table 5:** Interaction between AF and polypharmacy on the risk of PIMs.

Stratum	Medication count	PIMs n/N (%)	aOR (95% CI)	Risk difference (%)	Interaction
No AF	<7 drugs	40/127 (31.5)	1.00 (Ref.)	–	*P* = .031
No AF	≥7 drugs	142/94 (65.2)	2.10 (1.33–3.31)	+33.7	
AF	<7 drugs	15/38 (39.5)	1.00 (Ref.)	–	
AF	≥7 drugs	212/150 (94.2)	5.12 (3.87–6.78)	+54.7	

AF = atrial fibrillation, aOR = adjusted odds ratio, CI = confidence interval, PIM = potentially inappropriate medication.

### 3.5. Sensitivity and robustness analyses

Three pre-specified sensitivity analyses confirmed the stability of our primary findings (Table 6). Excluding patients with CCU admission > 48 hours (n = 52) yielded minimal changes in effect estimates: the AF association attenuated from an adjusted OR of 2.98 to 2.91 (2.3% change), while polypharmacy remained virtually unchanged (adjusted odds ratio [aOR] = 3.41 vs 3.45). After excluding severe renal impairment (eGFR < 30 mL/min/1.73 m^2^, n = 38), the AF–PIMs association persisted robustly (aOR = 3.05), though the eGFR < 45 category lost statistical significance (aOR = 1.52, 95% CI: 0.88–2.63, *P* = .13), likely reflecting diminished statistical power and collinearity. Excluding patients with prior 30-day readmissions (n = 94) produced the most conservative estimates, yet all 3 core predictors remained significant with <10% change in effect sizes.

**Table 6:** Sensitivity analyses under various exclusion criteria.

Variable	Main model (n = 468)	Excluding CCU (n = 416)	% Change	Excluding eGFR < 30 (n = 430)	% Change	Excluding readmissions (n = 374)	% Change
Atrial fibrillation	2.98 (1.89–4.68)	2.91 (1.82–4.65)	−2.3%	3.05 (1.92–4.84)	+2.3%	2.84 (1.75–4.62)	−4.7%
Medications ≥ 7	3.45 (2.10–5.66)	3.41 (2.06–5.64)	−1.2%	3.38 (2.04–5.60)	−2.0%	3.21 (1.89–5.45)	−7.0%
e-GFR < 45 mL/min/1.73 m^2^	1.74 (1.05–2.88)	1.69 (0.99–2.89)	−2.9%	1.52 (0.88–2.63)	*	1.68 (0.96–2.94)	−3.4%

CCU = cardiac care unit, e-GFR = estimated glomerular filtration rate.

* Not applicable due to reduced sample size and collinearity.

### 3.6. Drug-specific PIMs analysis

Table 7 details the 14 cases of failed renal dose adjustment, representing 1.4% of all PIMs and 3.4% of the cohort. The most egregious omission was spironolactone prescribed at standard dose (20–40 mg daily) in patients with eGFR < 30 mL/min/1.73 m^2^ (n = 9), contravening Beers Criteria that mandate dose reduction or avoidance due to life-threatening hyperkalemia risk. Notably, all 9 patients had baseline serum potassium > 4.5 mmol/L and concurrent angiotensin-converting enzyme inhibitor/angiotensin receptor blocker use, yet potassium monitoring was documented in only 2 cases within 7 days post-discharge. Among direct oral anticoagulants (DOACs), Edoxaban 60 mg was inappropriately maintained in 3 patients with eGFR 15 to 30 mL/min, despite FDA label restrictions requiring dose reduction to 30 mg or avoidance. Renal dosing failures occurred in 14 patients (2.6% of the cohort), none of which triggered EMR alerts. This suggests potential blind spots in EMR decision support that warrant validation in larger samples.

**Table 7:** Cases of failed renal dose adjustment (n = 14).

Drug	Prescribed dose	eGFR (mL/min/1.73 m^2^)	n (%)	Beers criterion violated	Consequence risk
Spironolactone	20–40 mg daily	<30	9 (64.3)	Avoid or dose reduce	Severe hyperkalemia
Spironolactone	40 mg daily	30–44	2 (16.7)	Use with caution & monitor K^+^	Moderate hyperkalemia
Edoxaban	60 mg daily	15–29	3 (25.0)	Dose reduce to 30 mg	Major bleeding

e-GFR = estimated glomerular filtration rate.

Table 8 enumerates the ten most prevalent DDIs, with RAS inhibitor–spironolactone constituting the highest risk dyad (21.8% of the cohort, high severity), followed by SGLT2 inhibitor–loop diuretic combinations (9.3%, moderate severity). Rivaroxaban-based interactions accounted for 3 of the top ten dyads, underscoring the complexity of anticoagulation management in this population. Collectively, these drug-specific analyses reveal that PIMs cluster around 3 critical domains – hyperkalemia risk from RAS-MRA combinations, bleeding from anticoagulant interactions, and volume depletion from sodium-glucose cotransporter 2 inhibitor-diuretic synergies – providing clear, actionable targets for pharmacist-led medication reconciliation and EMR-based clinical decision support enhancements.

**Table 8:** Most prevalent drug–drug interactions in older adults with heart failure.

Rank	Drug combination	n (%)	Primary risk	Severity	Clinical notes
1	RAS inhibitor (ACEI/ARB/ARNI) + Spironolactone	89 (21.8)	Hyperkalemia	High	12 patients had concurrent potassium supplements or TMP-SMX
2	SGLT2 inhibitor + Loop diuretic	38 (9.3)	Volume depletion/ AKI	Moderate	Empagliflozin-furosemide most common
3	Rivaroxaban + Amiodarone	18 (4.4)	Bleeding (CYP3A4/P-gp)	High	3 cases with INR > 4.0
4	Warfarin + Amiodarone	7 (1.7)	Bleeding (CYP2C9)	High	3 cases supratherapeutic INR (>4.0) documented
5	Digoxin + Amiodarone	6 (1.5)	Digoxin toxicity	High	Serum level monitoring absent in 5/6 cases
6	NSAID + RAS inhibitor	5 (1.2)	Renal failure/ HF worsening	High	Ibuprofen or diclofenac; 3 patients eGFR < 45mL/min/1.73 m^2^
7	Aspirin + Warfarin	5 (1.2)	Bleeding	Moderate	No documented GI prophylaxis in 4/5 cases
8	SGLT2 inhibitor + Insulin	5 (1.2)	Euglycemic ketoacidosis	Moderate	All empagliflozin; basal insulin doses > 30 units
9	Potassium supplement + RAS inhibitor + Spironolactone (Triple)	4 (1.0)	Hyperkalemia	High	eGFR < 60 mL/min/1.73 m^2^; no enhanced monitoring
10	Clarithromycin + Rivaroxaban	3 (0.7)	Bleeding (CYP3A4)	High	All short-course (5–7 d) for respiratory infection

ACEI = angiotensin-converting enzyme inhibitor, ARB = angiotensin receptor blocker, ARNI = angiotensin receptor-neprilysin inhibitor, e-GFR = estimated glomerular filtration rate, HF = heart failure, SGLT2i = sodium-glucose cotransporter 2 inhibitor.

## 4. Discussion

We observed a PIM prevalence of 87.4% at discharge, substantially exceeding our hypothesis and establishing this transition as a critical vulnerability for older patients with HF. This figure contrasts with 53% reported in U.S. ambulatory HF clinics and 62 to 70% in Chinese outpatient cohorts.^[20–22]^ The 15 to 20 percentage-point escalation reflects the discharge transition risk, wherein hospitalization intensifies medication regimens while systematic reconciliation remains suboptimal. Our data reveal that polypharmacy accumulation is exponential: patients discharged with ≥7 medications faced a 75.2% probability of harboring at least one PIM, translating to a 3.45-fold adjusted risk compared to those on <7 drugs. This receiver operating characteristic-derived threshold identifies an actionable cut-point beyond which enhanced pharmacovigilance resources may be warranted.

The high PIM prevalence of 87.4% in this study is not an overestimation but reflects real-world complexity at discharge. This rate is higher than previous outpatient studies mainly because: we focused on the high-risk discharge transition where medication regimens are intensified; we included the Beers “Use with Caution” category; and all potential PIMs were manually reviewed by senior pharmacists with full consideration of clinical context (indication, dosage, renal function, and patient characteristics) to avoid false positives. Thus, the elevated PIM rate genuinely represents the prescribing vulnerability in this population.

Notably, mounting evidence has confirmed that PIM exposure is strongly associated with adverse clinical outcomes in older adults with HF. Previous meta-analyses have demonstrated that PIMs are linked to a 30% higher risk of 30-day hospital readmission, a 1.8-fold increased risk of all-cause mortality within 6 months, and a higher incidence of adverse drug events including falls, acute kidney injury, hyperkalemia, and bleeding complications. In our cohort, the 30-day readmission rate was numerically higher in the PIM-positive group (21.3% vs 11.9%), consistent with these outcome-associated risks. Although the present study was not designed to ascertain the causal impact of PIMs on hard clinical endpoints, the alarmingly high PIM prevalence and established prognostic implications strongly support the clinical urgency of reducing inappropriate prescribing at discharge to improve safety and outcomes in this vulnerable population.^[23,24]^

AF functioned as a potent predictor, driven by obligatory prescription of rate- and rhythm-control agents that inherently trigger Beers warnings. This vulnerability is magnified by the narrow therapeutic windows of antiarrhythmic agents (e.g., amiodarone, digoxin) and the renal clearance dependencies of DOACs, which require meticulous dose adjustment – processes frequently compromised during fragmented discharge transitions. Furthermore, AF obligates anticoagulation that interacts synergistically with HF therapies (spironolactone, SGLT2 inhibitors), creating exponential opportunities for drug-drug interactions and additive toxicity.^[25–27]^ The synergistic interaction we observed – wherein AF patients accumulating ≥7 medications faced a 5.12-fold escalated PIM risk versus 2.10-fold in non-AF patients – underscores that polypharmacy effects are amplified by arrhythmia-related prescribing complexity.

Building on these disease-specific predictors, our effect size comparison reveals important distinctions: renal impairment’s association (aOR = 1.74, 95% CI: 1.05–2.88) is quantitatively modest relative to polypharmacy (aOR = 3.45) and AF (aOR = 2.98). The 95% CI lower bound approaching unity (1.05) reflects statistical fragility, likely due to sample size constraints. We, therefore, characterize it as an independent, moderate-strength predictor rather than a component of a “synergistic triad.”

Our drug-specific analysis further clarifies the mechanism: rather than generic under-dosing, we found complete omission of evidence-based adjustments – 68.1% of spironolactone PIMs violated eGFR < 45 dosing thresholds, and 3 Edoxaban patients with eGFR 15 to 29 mL/min received full 60 mg doses despite black-box warnings. Notably, none of these 14 renal dosing failures triggered automated pharmacist consultation. These observations suggest potential gaps in EMR-based clinical decision support for high-risk medications, particularly renally cleared drugs with narrow therapeutic windows. However, the small sample size (n = 14, 2.6% of cohort) precludes conclusions about systemic failure and highlights the need for prospective validation across multiple centers.^[28]^

The standard Charlson index’s VIF of 2.8 reflected the HF weight ceiling effect, masking comorbidity contributions. Our pre-specified modified CCI (excluding HF weight) revealed a stronger, statistically significant association (aOR = 1.67, 95% CI: 1.12–2.48, VIF = 1.4) that was concealed in the primary model. Although m-CCI resolves multicollinearity, its clinical interpretability requires caution – removing HF weight may underestimate severity in HF-dominant patients. Future studies should validate m-CCI against external HF cohorts.^[29,30]^

These findings collectively yield 3 potential intervention targets. First, the top 3 DDI dyads – RAS inhibitor–spironolactone (21.8%), SGLT2 inhibitor–loop diuretic (9.3%), and rivaroxaban–amiodarone (4.4%) – account for >35% of high-severity PIMs and could trigger pharmacy review. Second, renal dosing failures suggest that EMR-integrated alerts might be considered for spironolactone and DOACs when eGFR falls below label-specified thresholds. Third, the ≥7 medication threshold could serve as a flag for enrollment in post-discharge reconciliation clinics. However, these inferences are limited by our study design and require prospective validation.

### 4.1. Limitations

Several limitations should be acknowledged. First, this was a single-center, retrospective observational study conducted at a tertiary hospital in Eastern China, which may restrict the generalization of our findings to other regions or primary care settings with limited pharmacy support and electronic medical record systems. Second, we only evaluated the prevalence and risk factors of PIMs at discharge, without collecting data on post-discharge clinical outcomes including readmission, adverse drug events, or mortality. Therefore, we could not establish a causal link between PIMs and poor clinical prognosis. Third, the 2023 AGS Beers Criteria used in this study were developed based on Western populations, and their applicability to elderly Asian patients with different pharmaceutical characteristics and clinical practice patterns requires further validation. Fourth, residual confounding may exist due to unmeasured geriatric domains including frailty, cognitive status, and functional capacity, which are closely associated with prescribing patterns and PIM risk in older adults. Finally, the relatively small sample of renal dosing failures limited our ability to draw definitive conclusions about system-level deficiencies in electronic alerting functions.

## 5. Conclusion

In conclusion, PIMs at discharge were highly prevalent among older adults with chronic HF in Eastern China, affecting 87.4% of patients. Multivariable analysis identified 3 independent risk factors: discharge medication count ≥ 7, AF, and renal impairment (eGFR < 45 mL/min/1.73 m^2^). A synergistic interaction between AF and polypharmacy further amplified the risk of PIMs. The most frequent PIMs were furosemide, rabeprazole, and rivaroxaban, while RAS inhibitor–spironolactone was the most common high-severity DDI.

These results have important clinical significance. The 3 readily identifiable risk factors – polypharmacy (≥7 drugs), AF, and renal dysfunction – can be used as straightforward warning indicators to prioritize pharmacist-led medication review at discharge. Integrating Beers Criteria into electronic medical record systems and implementing targeted medication reconciliation for high-risk patients may effectively reduce PIM-related harm, improve medication safety, and optimize pharmacotherapy in this vulnerable elderly population.

## Acknowledgments

Junfen Xu served as first author, conceptualized the study, performed the statistical analysis, and drafted the manuscript. Liqin Zhang extracted and validated the medication data, contributed to methodology design, and revised the manuscript. Hailiang Ma adjudicated potentially inappropriate medications and supervised data quality control. Yuanben Lu collected clinical data and coordinated the retrospective chart review. Zhenhua Jiang, as corresponding author, provided overall project supervision, and critically revised the manuscript for intellectual content. All authors approved the final manuscript submission.

## Author contributions

**Conceptualization:** Zhenhua Jiang.

**Data curation:** Liqin Zhang.

**Formal analysis:** Liqin Zhang, Hailiang Ma.

**Investigation:** Hailiang Ma.

**Methodology:** Hailiang Ma, Yuanben Lu.

**Resources:** Yuanben Lu.

**Software:** Yuanben Lu.

**Validation:** Zhenhua Jiang.

**Writing – original draft:** Junfen Xu.

**Writing – review & editing:** Zhenhua Jiang.

## References

[1] GBD 2021 Risk Factors Collaborators. Global burden and strength of evidence for 88 risk factors in 204 countries and 811 subnational locations, 1990–2021: a systematic analysis for the Global Burden of Disease Study 2021. Lancet. 2024;403:2162–203. 38762324 10.1016/S0140-6736(24)00933-4 PMC11120204

[2] Liu S Li Y Zeng X. Burden of cardiovascular diseases in China, 1990–2016: findings from the 2016 Global Burden of Disease Study. JAMA Cardiol. 2019;4:342–52. 30865215 10.1001/jamacardio.2019.0295 PMC6484795

[3] Feng J Zhang Y Zhang J. Epidemiology and burden of heart failure in Asia. JACC Asia. 2024;4:249–64. 38660101 10.1016/j.jacasi.2024.01.013 PMC11035951

[4] Bhagavathula AS Gebreyohannes EA Fialova D. Prevalence of polypharmacy and risks of potentially inappropriate medication use in the older population in a developing country: a systematic review and meta-analysis. Gerontology. 2022;68:136–45. 33975303 10.1159/000516075

[5] Muhlack DC Hoppe LK Weberpals J Brenner H Schöttker B. The association of potentially inappropriate medication at older age with cardiovascular events and overall mortality: a systematic review and meta-analysis of cohort studies. J Am Med Dir Assoc. 2017;18:211–20. 28131719 10.1016/j.jamda.2016.11.025

[6] Rado J Janicak PG. Pharmacological and clinical profile of recently approved second-generation antipsychotics: implications for treatment of schizophrenia in older patients. Drugs Aging. 2012;29:783–91. 23018584 10.1007/s40266-012-0009-0

[7] Fauchier L Bisson A Bodin A. Heart failure with preserved ejection fraction and atrial fibrillation: recent advances and open questions. BMC Med. 2023;21:54. 36782248 10.1186/s12916-023-02764-3 PMC9926737

[8] Oraii A Mcintyre WF Parkash R. Atrial fibrillation ablation in heart failure with reduced vs preserved ejection fraction: a systematic review and meta-analysis. JAMA Cardiol. 2024;9:545–55. 38656292 10.1001/jamacardio.2024.0675 PMC11044015

[9] Yadesa TM Kitutu FE Deyno S Ogwang PE Tamukong R Alele PE. Prevalence, characteristics and predicting risk factors of adverse drug reactions among hospitalized older adults: a systematic review and meta-analysis. SAGE Open Med. 2021;9:20503121211039099. 34422271 10.1177/20503121211039099 PMC8377309

[10] Zhao D Liu J Wang M Zhang X Zhou M. Epidemiology of cardiovascular disease in China: current features and implications. Nat Rev Cardiol. 2019;16:203–12. 30467329 10.1038/s41569-018-0119-4

[11] Noordzij M Tripepi G Dekker FW Zoccali C Tanck MW Jager KJ. Sample size calculations: basic principles and common pitfalls. Nephrol Dial Transplant. 2010;25:1388–93. 20067907 10.1093/ndt/gfp732

[12] Charlson ME Pompei P Ales KL MacKenzie CR. A new method of classifying prognostic comorbidity in longitudinal studies: development and validation. J Chronic Dis. 1987;40:373–83. 3558716 10.1016/0021-9681(87)90171-8

[13] Bennett CC. Utilizing RxNorm to support practical computing applications: capturing medication history in live electronic health records. J Biomed Inform. 2012;45:634–41. 22426081 10.1016/j.jbi.2012.02.011

[14] Greene SJ Butler J Albert NM. Medical therapy for heart failure with reduced ejection fraction: the CHAMP-HF registry. J Am Coll Cardiol. 2018;72:351–66. 30025570 10.1016/j.jacc.2018.04.070

[15] Bajic B Galic I Mihailovic N. Performance of Charlson and Elixhauser comorbidity index to predict in-hospital mortality in patients with stroke in Sumadija and Western Serbia. Iran J Public Health. 2021;50:970–7. 34183955 10.18502/ijph.v50i5.6114 PMC8223561

[16] Jaber D Vargas F Nguyen L. Prescriptions for potentially inappropriate medications from the Beers Criteria among older adults hospitalized for heart failure. J Card Fail. 2022;28:906–15. 34818566 10.1016/j.cardfail.2021.11.014 PMC9344978

[17] Abraham NS Hlatky MA Antman EM; ACCF/ACG/AHA. ACCF/ACG/AHA 2010 expert consensus document on the concomitant use of proton pump inhibitors and thienopyridines: a focused update of the ACCF/ACG/AHA 2008 expert consensus document on reducing the gastrointestinal risks of antiplatelet therapy and NSAID use. Am J Gastroenterol. 2010;105:2533–49. 21131924 10.1038/ajg.2010.445

[18] Moudallel S Van Laere S Cornu P Dupont A Steurbaut S. Assessment of adherence, treatment satisfaction and knowledge of direct oral anticoagulants in atrial fibrillation patients. Br J Clin Pharmacol. 2022;88:2419–29. 34907577 10.1111/bcp.15180

[19] Kowey PR Reiffel JA Ellenbogen KA Naccarelli GV Pratt CM. Efficacy and safety of prescription omega-3 fatty acids for the prevention of recurrent symptomatic atrial fibrillation: a randomized controlled trial. JAMA. 2010;304:2363–72. 21078810 10.1001/jama.2010.1735

[20] Bermingham M Ryder M Travers B. The St Vincent’s potentially inappropriate medicines study: development of a disease-specific consensus list and its evaluation in ambulatory heart failure care. Eur J Heart Fail. 2014;16:915–22. 25100110 10.1002/ejhf.132

[21] Liu CL Peng LN Chen YT Lin M-H Liu L-K Chen L-K. Potentially inappropriate prescribing (IP) for elderly medical inpatients in Taiwan: a hospital-based study. Arch Gerontol Geriatr. 2012;55:148–51. 21820189 10.1016/j.archger.2011.07.001

[22] Wang F Ma Z Liu M Wu X. Potentially inappropriate medications at admission and discharge in older adults: a comparison of the Beers 2019 and 2015 Criteria. Int J Clin Pharmacol Ther. 2020;58:299–309. 32301700 10.5414/CP203638

[23] Muzzarelli I Neumeier VI Gagesch M Rösler W Burch AR. Association of potentially inappropriate medications with rehospitalisation and death within three months in older patients: a systematic review and meta-analysis. Int J Clin Pharm. 2026;48:350–63. 40996586 10.1007/s11096-025-02013-y PMC12992462

[24] Li M Wei N Shi HY. Prevalence and clinical implications of polypharmacy and potentially inappropriate medication in elderly patients with heart failure: results of six months’ follow-up. J Geriatr Cardiol. 2023;20:495–508. 37576481 10.26599/1671-5411.2023.07.002 PMC10412538

[25] Jang SM Bahjri K Tran H. Safety and efficacy of direct oral anticoagulants for atrial fibrillation in patients with renal impairment. Pharmacy (Basel). 2020;8:30. 32143504 10.3390/pharmacy8010030 PMC7151721

[26] Zaki HA Bashir K Iftikhar H. An integrative comparative study between digoxin and amiodarone as an emergency treatment for patients with atrial fibrillation with evidence of heart failure: a systematic review and meta-analysis. Cureus. 2022;14:e26800. 35971374 10.7759/cureus.26800 PMC9372377

[27] Savarese G Giugliano RP Rosano GM. Efficacy and safety of novel oral anticoagulants in patients with atrial fibrillation and heart failure: a meta-analysis. JACC Heart Fail. 2016;4:870–80. 27614940 10.1016/j.jchf.2016.07.012

[28] Mccoy AB Waitman LR Gadd CS. A computerized provider order entry intervention for medication safety during acute kidney injury: a quality improvement report. Am J Kidney Dis. 2010;56:832–41. 20709437 10.1053/j.ajkd.2010.05.024 PMC2963668

[29] Charlson ME Carrozzino D Guidi J Patierno C. Charlson comorbidity index: a critical review of clinimetric properties. Psychother Psychosom. 2022;91:8–35. 34991091 10.1159/000521288

[30] Austin PC. Goodness-of-fit diagnostics for the propensity score model when estimating treatment effects using covariate adjustment with the propensity score. Pharmacoepidemiol Drug Saf. 2008;17:1202–17. 18972454 10.1002/pds.1673

